# Laparoscopic Transabdominal Pre-peritoneal Repair of a Bilateral Inguinal Hernia in a Pediatric Female Patient in Pakistan: A Case Report

**DOI:** 10.7759/cureus.54186

**Published:** 2024-02-14

**Authors:** Arsalan Baig, Murk Lakhani, Shajie Ur Rehman Usmani

**Affiliations:** 1 General Surgery, Dr. Ruth Pfau Hospital, Karachi, PAK; 2 Surgery, Dow University of Health Sciences, Karachi, PAK; 3 Surgery, Civil Hospital Karachi, Karachi, PAK; 4 Pediatric Surgery, Dow University of Health Sciences, Karachi, PAK

**Keywords:** pakistan, pediatric, female, inguinal hernia, bilateral, laparoscopic repair, tapp

## Abstract

Inguinal hernias, although a common occurrence, pose a significant threat to the surgical community on account of their complexity and socioeconomic consequences. Bilateral inguinal hernias, which are a rare subtype of inguinal hernias, in particular, are problematic since there are no existing definitive international guidelines for their repair. It is estimated that between 8% and 30% of inguinal hernia patients have bilateral hernias, but there is still no clarity as to whether a bilateral hernia represents a special type of inguinal hernia or two different hernias in one patient. The transabdominal pre-peritoneal repair (TAPP), totally extra-peritoneal repair (TEP), and Lichtenstein repair techniques are commonly employed depending on the resources and surgical expertise available, but there is a need to conduct large-scale, prospective, randomized-controlled trials to guide the formation of evidence-based guidelines that could be followed globally. Herein, we present the first known case of a bilateral inguinal hernia in a female pediatric patient repaired by the laparoscopic TAPP technique from Pakistan.

## Introduction

An inguinal hernia (IH) is one of the greatest challenges in surgical pathology because of its complexity, frequency, as well as socioeconomic consequences. The lifetime occurrence of groin hernia (GH), which includes both IH and femoral hernia, is 27-43% in men and 3-6% in women [1,2]. IH is mostly symptomatic and is usually diagnosed by clinical history and examination. The content of the hernial sac may be determined by imaging, particularly through ultrasound and computed tomography. Surgery is the only curative therapy for IH [3,4]. The new International Guidelines of the HerniaSurge Group only recommend the transabdominal pre-peritoneal repair (TAPP), totally extraperitoneal repair (TEP), and Lichtenstein repair techniques for IH.

A special entity among inguinal hernias is represented by bilateral hernias and only a few studies in the literature have focused specifically on bilateral IH [5,6]. Bilateral IH is rarer in females as compared to males, and it is estimated that 8-30% of hernia patients have bilateral hernias, but there is still no clarity as to whether a bilateral hernia represents a special type of IH or two separate hernias in a single patient. Herein, we present the first known case of a bilateral IH in a female pediatric patient repaired by the laparoscopic TAPP technique from Pakistan.

## Case presentation

A 12-year-old female patient presented to a tertiary care hospital with a complaint of bilateral inguinal swellings since childhood. The swellings were gradually increasing in size and reducible and became more obvious when she strained, coughed, or assumed an upright position. There was no history of chronic cough, constipation, and urinary straining. The per-abdominal examination was unremarkable. The inguinal region exhibited bilateral inguinal swellings of 2.3 cm on the left side and 1.2 cm on the right side, with normal overlying skin and ill-defined edges. They were soft, non-tender, non-warm, and reducible. Ultrasound scan detected a defect of 1.2 cm in the left inguinal region and 0.8 cm in the right inguinal region, suggestive of bilateral IH.

**Figure 1:**
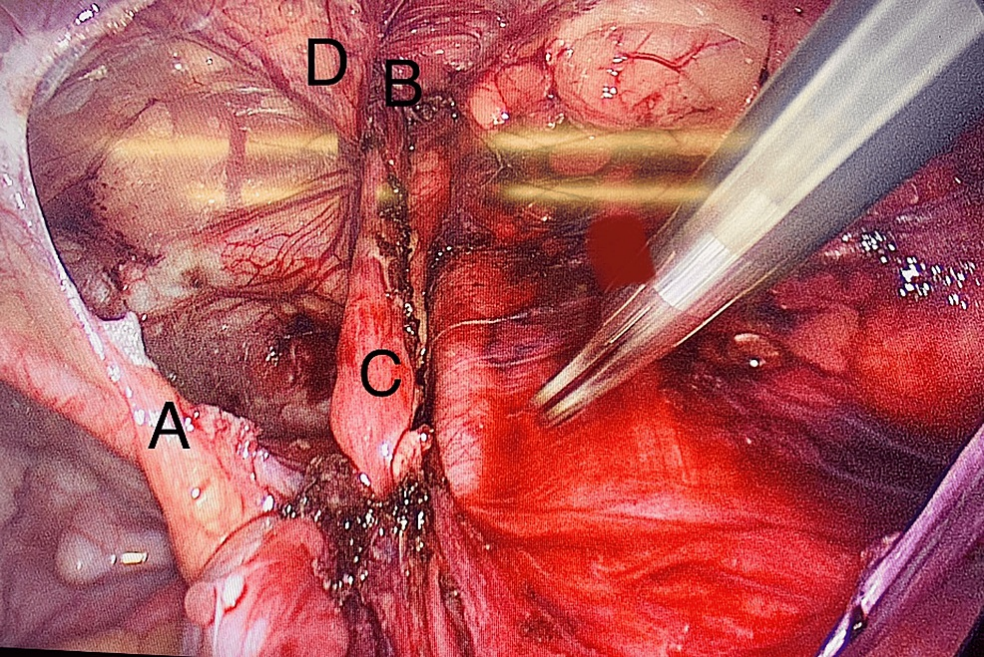
Indirect inguinal hernia containing a round ligament A, Medial umbilical ligament; B, Deep inguinal ring; C, Round ligament of uterus; D, Inferior epigastric vessels

After taking written, informed consent, bilateral laparoscopic TAPP repair was performed, which revealed a bilateral indirect IH containing the round ligament (Figure 1). Peritoneal flaps were raised 5 cm above and below the hernia defect. A prolene mesh was placed over the everted space and tagged with a tacker in order to reduce the recurrence rate. The peritoneal flaps were closed with a running non-absorbable suture. A similar procedure was done for the other defect. The postoperative period was mainly uneventful. The patient was discharged the next day and remained stable with no active issues on the follow-up visits.

## Discussion

**Figure 2:**
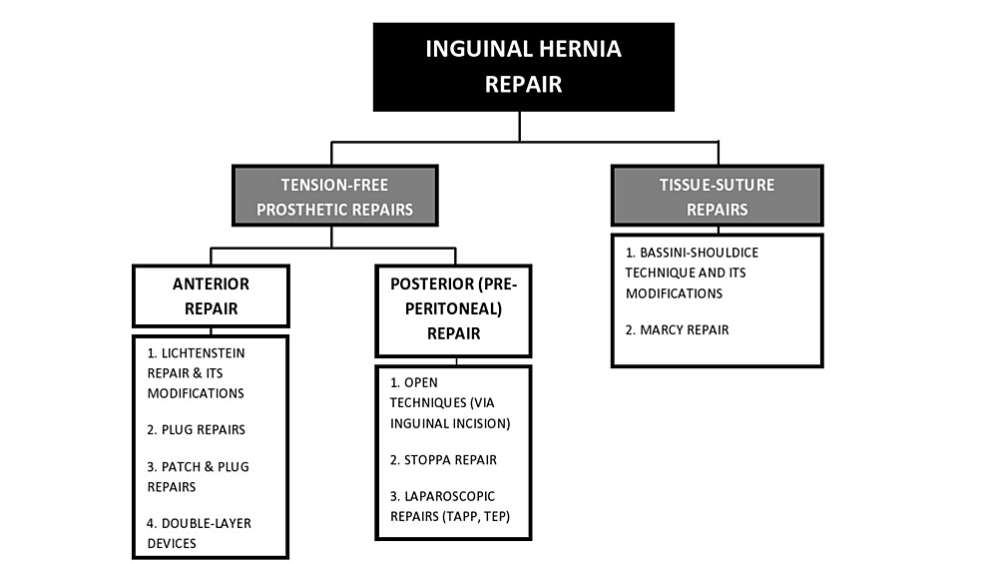
Summary of repair techniques for inguinal hernia TAPP, Transabdominal pre-peritoneal; TEP, Total extra-peritoneal

IH is comparatively rarer in females than males, comprising 2%-11.5% of all hernias [7]. This is due to a relatively narrower inguinal canal in females along with a wider rectus abdominis muscle. Apart from the typical symptoms of groin swelling, about one-third of the patients with IH present with a hernial complication such as abdominal pain, tenderness, irreducible hernia, and enterocutaneous fistula [8]. Out of all IH surgical repair techniques (Figure 2), the International Guidelines for GH Management recommend laparo-endoscopic repair for the treatment of primary bilateral IH, provided that surgeons with sufficient expertise and specific resources are available [1]; otherwise, the open technique is used. The TAPP and TEP approaches for IH so far have shown similar outcomes in terms of hospital stay, recovery, chronic pain, and quality of life [9]. TAPP is easier to perform; however, it has a longer operating time and a greater incidence of postoperative pain. On the other hand, TEP is reportedly associated with a greater incidence of seroma formation. A study reported that the risk for both seroma and hematoma formation is also comparable amongst TEP, TAPP, and open repair [10]. In our case, TAPP was performed based on local practices and the expertise of the surgeons involved.

While international guidelines exist for unilateral IH, no official recommendations exist for bilateral IH. However, the European Hernia Society has pointed out that laparoscopic repair of bilateral hernias is associated with improved short-term patient outcomes without compromising on long-term outcomes [3,11]. This is the primary reason why TAPP has been preferred in our local practices despite the lack of definitive guidelines. Moreover, TAPP has shown favorable outcomes for patients with unilateral IH in our setup, making it the wiser choice.

Only three randomized prospective trials (RCTs) compare laparoscopic repair of bilateral inguinal hernias to open repair. Sarli et al. demonstrated that laparoscopic repair was associated with faster recovery and decreased postoperative pain while days of hospitalization, complications, and recurrence rates were similar in both groups [12]. These results were subsequently supported by Mahon et al. in their study [13]. The third and last RCT by Ielpo et al. also seconded the results of the prior RCTs in terms of beneficial short-term results in laparoscopic repair, including recovery, postoperative pain, and complications [14]. However, all of the aforementioned trials included only a small number of patients, hence diminishing the credibility of the results. Also, only one large-scale study has deemed laparoscopic repair to be at least non-inferior to open repair and that it should be considered the ‘gold standard’ [15]. Given that this study was retrospective and non-randomized, the level of evidence is insufficient to establish a ‘gold standard’, nevertheless, it still provides an indication. Since our patient had an uncomplicated postoperative period, it is reasonable to support the prior statement and pursue the laparoscopic approach while we wait for definitive evidential guidelines.

## Conclusions

TAPP repair for bilateral IH is a safe and reasonable option in terms of both short-term and long-term outcomes. Importantly, there is a need for high-quality prospective randomized studies to establish evidence-based guidelines for the definitive treatment of bilateral IH in order to streamline clinical practice and improve clinical outcomes universally. Finally, there is a further need to investigate the differences in the clinical presentation of IH and the efficacy of different surgical repairs for IH in women and men separately.

## References

[1] The HerniaSurge Group (2018). International guidelines for groin hernia management. Hernia.

[2] Kingsnorth A, LeBlanc K (2003). Hernias: inguinal and incisional. Lancet.

[3] Simons MP, Aufenacker T, Bay-Nielsen M (2009). European Hernia Society guidelines on the treatment of inguinal hernia in adult patients. Hernia.

[4] Konschake M, Zwierzina M, Moriggl B (2020). The inguinal region revisited: the surgical point of view. An anatomical-surgical mapping and sonographic approach regarding postoperative chronic groin pain following open hernia repair. Hernia.

[5] Fischer S, Cassivi S, Paul A (1999). Evidence-based medicine and special aspects in bilateral inguinal hernia repair. Hernia.

[6] Frankum CE, Ramshaw BJ, White J (1999). Laparoscopic repair of bilateral and recurrent hernias. Am Surg.

[7] Chawla S (2001). Inguinal hernia in females. Med J Armed Forces India.

[8] Ohene-Yeboah M, Abantanga FA (2011). Inguinal hernia disease in Africa: a common but neglected surgical condition. West Afr J Med.

[9] Trindade EN, Trindade MR (2011). The best laparoscopic hernia repair. TEP or TAPP?. Ann Surg.

[10] Aiolfi A, Cavalli M, Micheletto G (2019). Primary inguinal hernia: systematic review and Bayesian network meta-analysis comparing open, laparoscopic transabdominal preperitoneal, totally extraperitoneal, and robotic preperitoneal repair. Hernia.

[11] Ramanan B, Maloley BJ, Fitzgibbons RJ Jr (2014). Inguinal hernia: follow or repair?. Adv Surg.

[12] Sarli L, Iusco DR, Sansebastiano G, Costi R (2001). Simultaneous repair of bilateral inguinal hernias: a prospective, randomized study of open, tension-free versus laparoscopic approach. Surg Laparosc Endosc Percutan Tech.

[13] Mahon D, Decadt B, Rhodes M (2003). Prospective randomized trial of laparoscopic (transabdominal preperitoneal) vs open (mesh) repair for bilateral and recurrent inguinal hernia. Surg Endosc.

[14] Ielpo B, Duran H, Diaz E (2018). A prospective randomized study comparing laparoscopic transabdominal preperitoneal (TAPP) versus Lichtenstein repair for bilateral inguinal hernias. Am J Surg.

[15] Wauschkuhn CA, Schwarz J, Boekeler U, Bittner R (2010). Laparoscopic inguinal hernia repair: gold standard in bilateral hernia repair? Results of more than 2800 patients in comparison to literature. Surg Endosc.

